# Genetic Evidence Supporting Fibroblast Growth Factor 21 Signalling as a Pharmacological Target for Cardiometabolic Outcomes and Alzheimer’s Disease

**DOI:** 10.3390/nu13051504

**Published:** 2021-04-29

**Authors:** Susanna C. Larsson, Dipender Gill

**Affiliations:** 1 Department of Surgical Sciences, Uppsala University, 751 85 Uppsala, Sweden; 2 Unit of Cardiovascular and Nutritional Epidemiology, Institute of Environmental Medicine, Karolinska Institutet, 171 77 Stockholm, Sweden; 3 Department of Epidemiology and Biostatistics, School of Public Health, St. Mary′s Hospital, Imperial College London, London W2 1PG, UK; dipender.gill@imperial.ac.uk; 4 Clinical Pharmacology and Therapeutics Section, Institute of Medical and Biomedical Education and Institute for Infection and Immunity, St. George’s, University of London, London SW17 0QT, UK; 5 Clinical Pharmacology Group, Pharmacy and Medicines Directorate, St. George’s University Hospitals NHS Foundation Trust, London SW17 0QT, UK; 6 Novo Nordisk Research Centre Oxford, Old Road Campus, Oxford OX3 7FZ, UK

**Keywords:** alcohol, cardiovascular disease, fibroblast growth factor 21, macronutrients, sugar, mendelian randomization

## Abstract

Fibroblast growth factor 21 (FGF21) is a human metabolic hormone whose effects include modification of macronutrient preference and energy homeostasis. In animal models, FGF21 has been shown to have beneficial effects on cardiometabolic outcomes, Alzheimer’s disease risk and lifespan. In this study, the single-nucleotide polymorphism rs838133 in the *FGF21* gene region was leveraged to investigate the potential clinical effects of targeting FGF21. The *FGF21* G allele was associated with lower intakes of total sugars and alcohol, and higher intakes of protein and fat as well as favourable with lipid levels, blood pressure traits, waist-to-hip ratio, systemic inflammation, cardiovascular outcomes, Alzheimer’s disease risk and lifespan. These findings may be used to anticipate the effects of pharmacologically increasing FGF21 signalling.

## 1. Introduction

Fibroblast growth factor 21 (FGF21) is a human metabolic hormone that is expressed in the liver [1]. The effects of FGF21 include altering of macronutrient preference and energy homeostasis [1,2,3]. Increased circulating FGF21 decreases the consumption of sweets, sugar and alcohol [2,4,5], and indirectly increases protein intake by suppressing sugar consumption [6]. In rodents and non-human primates, FGF21 treatment has beneficial effects on cardiometabolic outcomes, such as reduction in fat mass and alleviation of hyperglycaemia, insulin resistance, dyslipidaemia, and cardiovascular diseases [7]. Furthermore, FGF21 has been implicated in protecting against Alzheimer’s disease [8], as well as improving lifespan [9]. However, studies investigating the association between circulating FGF21 and these clinical outcomes in humans have been inconclusive [7,10]. To investigate this further, we aimed to leverage human genetic data within the Mendelian randomization paradigm to investigate the associations of a common allele in the *FGF21* gene with cardiometabolic outcomes, Alzheimer’s disease and lifespan [11].

## 2. Materials and Methods

We used the single-nucleotide polymorphism rs838133 in the *FGF21* gene region, previously shown to be associated with intake of macronutrients, alcohol and sweets [10,12], to assess potential effects of increasing FGF21 concentrations. Publicly available summary-level data for the associations of the *FGF21* variant with macronutrient and alcohol intake and other outcomes were obtained from the UK Biobank cohort and genome-wide association study consortia [13,14,15,16,17,18,19,20,21,22,23,24]. Details of the outcome datasets used in this study are provided in Table S1. For cardiometabolic diseases, we meta-analysed the estimates from the different data sources using inverse-variance models with fixed effects.

Only summary-level (i.e., aggregated) data were analysed in this study, for which appropriate ethical approval and participant consent had previously been obtained. The present Mendelian randomization analyses were approved by the Swedish Ethical Review Authority. We confirm that we have read the Journal’s position on issues involved in ethical publication and affirm that this work is consistent with those guidelines.

## 3. Results

**Figure 1:**
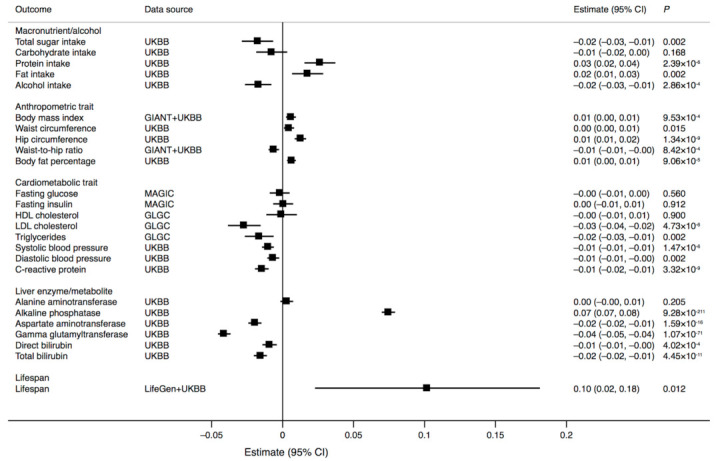
Associations of the *FGF21* rs838133 G allele with macronutrient intake, anthropometric and cardiometabolic traits, liver enzymes and metabolites, and lifespan. The outcomes are in standard deviation units except for lifespan (in years). CI: confidence interval.

The G (major) allele of rs838133 in the *FGF21* gene region was associated with lower intakes of total sugars and alcohol, and higher intakes of protein and fat (Figure 1), consistent with the expected effect of an increase in FGF21 concentration. We scaled all results per additional G allele to mimic the effect of elevated FGF21 concentrations.

The *FGF21* rs838133 G allele was associated with greater body mass index, body fat percentage and waist and hip circumferences, but with lower waist-to-hip ratio (Figure 1). Additionally, the G allele was associated with lower low-density lipoprotein cholesterol and triglyceride concentrations, lower systolic and diastolic blood pressure, and lower C-reactive protein concentrations, but was not associated with fasting glucose or fasting insulin concentrations (Figure 1). There was a positive association of the *FGF21* rs838133 G allele with the liver enzyme alkaline phosphatase, but a negative association with aspartate aminotransferase, gamma glutamyltransferase, and direct and total bilirubin concentrations (Figure 1). There was suggestive evidence of a positive association between the *FGF21* rs838133 G allele and lifespan (based on parental lifespan) (Figure 1).

**Figure 2:**
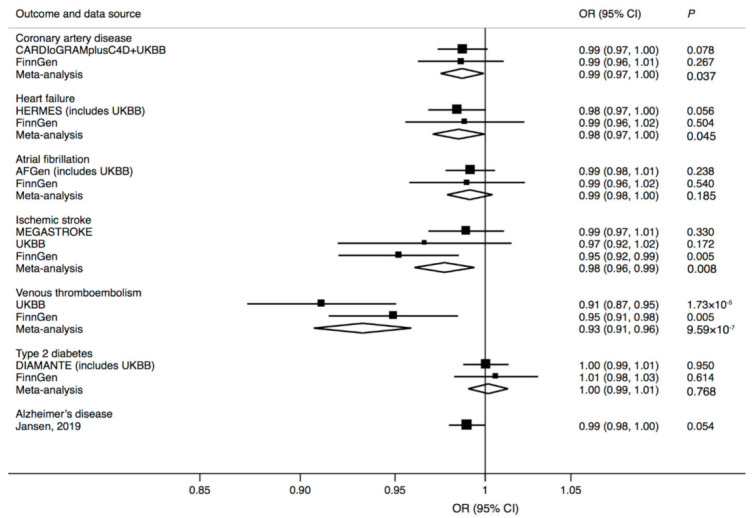
Associations of the *FGF21* rs838133 G allele with cardiometabolic diseases and Alzheimer’s disease. CI: confidence interval; OR: odds ratio.

In analyses of cardiovascular diseases, the *FGF21* rs838133 G allele was strongly associated with a reduced risk of venous thromboembolism, and had suggestive inverse associations with coronary artery disease, heart failure, and ischemic stroke (Figure 2). There was a suggestive association of the *FGF21* rs838133 G allele with reduced risk of Alzheimer’s disease (based on clinically diagnosed Alzheimer’s disease and Alzheimer’s disease by proxy cases and their corresponding controls), but no association with type 2 diabetes (Figure 2).

## 4. Discussion

This work leveraged human genetic data to provide insight into the broad metabolic and clinical effects of the major (G) allele of rs838133 in the *FGF21* gene. Our results support previously reported associations between the *FGF21* rs838133 variant with macronutrient and alcohol intake, lipid levels, blood pressure, waist-to-hip ratio, and liver enzymes as well as the lack of association with type 2 diabetes in the UK Biobank cohort [10]. It should be noted that the previous study used the minor (A) allele of the rs838133 variant as the effect allele [10] and thus showed associations in the opposite direction to our findings. The present study went further to provide novel evidence that the major allele of rs838133 in the *FGF21* gene was associated with decreased systemic inflammation (estimated by C-reactive protein concentration). We also identified a potential beneficial effect of the major allele at *FGF21* rs838133 on cardiovascular outcomes, with the strongest association for venous thromboembolism, as well as on Alzheimer’s disease and lifespan.

These findings inform on the potential effects of pharmacologically increasing FGF21 concentrations or signalling. The limitations of this study should also be acknowledged. Critically, it is possible that some of the identified associations may be attributable to genetic confounding, where the *FGF21* genetic variant also has pleiotropic associations unrelated to FGF21. As summary genetic association data for circulating FGF21 concentrations were not available, we could not perform colocalization analysis to explore this possibility [25]. Furthermore, the outcomes that we studied were determined by the availability of corresponding large-scale genetic association summary data. As such, it was not possible to perform analyses for other relevant traits, such as non-alcoholic steatohepatitis.

## 5. Conclusions

In summary, we used a major allele of rs838133 in the *FGF21* gene to identify evidence for its effects on macronutrient and alcohol intake as well as favourable effects on a range of cardiometabolic outcomes, Alzheimer’s disease and lifespan. This work anticipates the effects of pharmacologically increasing FGF21 signalling.

## Data Availability

All data used in this study are publicly available summary statistics data, with relevant data available from cited studies. The summary statistics data analyzed in this study are available on request from the corresponding author.

## Supplementary Materials

The following are available online at https://www.mdpi.com/article/10.3390/nu13051504/s1, Table S1: Data sources for the outcome phenotypes included in the present Mendelian randomization study.
