# Supplementary material for: Genetic Evidence Supporting Fibroblast Growth Factor 21 Signalling as a Pharmacological Target for Cardiometabolic Outcomes and Alzheimer’s Disease
Source: Nutrients. 2021 Apr 29;13(5):1504. doi: 10.3390/nu13051504 (PMC8146158; doi:10.3390/nu13051504)
Supplement: Supplementary file 1 [file nutrients-13-01504-s001.zip › nutrients-1160299-supplementary.pdf]

Table S1. Data sources for the outcome phenotypes included in the present Mendelian randomization study.

Outcome phenotype*	ID for the outcomes obtained through the MR Base platform or name of the outcome in FinnGen	Study or consortium	Sample size or cases/controls	First author and reference
Macronutrient/alcohol				
Total sugar intake†	ukb-b-17079; 100008: Output from GWAS pipeline using Phesant derived variables from UKBB (MRC-IEU)	UKBB	64,979	Hemani, 2018 [15]
Carbohydrate intake†	ukb-b-7244; 100005: Output from GWAS pipeline using Phesant derived variables from UKBB (MRC-IEU)	UKBB	64,979	Hemani, 2018 [15]
Protein intake†	ukb-b-12043; 100003: Output from GWAS pipeline using Phesant derived variables from UKBB (MRC-IEU)	UKBB	64,979	Hemani, 2018 [15]
Fat intake†	ukb-b-12379: 100004: Output from GWAS pipeline using Phesant derived variables from UKBB (MRC-IEU)	UKBB	64,979	Hemani, 2018 [15]
Alcohol intake†	ukb-b-5359; 100022: Output from GWAS pipeline using Phesant derived variables from UKBB (MRC-IEU)	UKBB	64,979	Hemani, 2018 [15]
Anthropometric trait				
Body mass index	NA	GIANT+UKBB	708,052	Pulit, 2019 [20]
Waist circumference	ukb-b-9405; 48: Output from GWAS pipeline using Phesant derived variables from UKBB (MRC-IEU)	UKBB	462,166	Hemani, 2018 [15]
Hip circumference	ukb-b-15590; 49: Output from GWAS pipeline using Phesant derived variables from UKBiobank (MRC-IEU)	UKBB	462,117	Hemani, 2018 [15]
Waist-to-hip ratio	NA	GIANT+UKBB	619,363	Pulit, 2019 [20]
Body fat percentage	ukb-b-8909; 23099: Output from GWAS pipeline using Phesant derived variables from UKBB (MRC-IEU)	UKBB	454,633	Hemani, 2018 [15]
Cardiometabolic trait				
Fasting glucose	ebi-a-GCST005186	MAGIC	58,074	Manning, 2012 [13]

Fasting insulin	ebi-a-GCST005185	MAGIC	51,750	Manning, 2012 [13]
Triglycerides	ieu-a-302	GLGC	177,861	Willer, 2013 [14]
LDL cholesterol	ieu-a-300	GLGC	173,082	Willer, 2013 [14]
HDL cholesterol	ieu-a-299	GLGC	187,167	Willer, 2013 [14]
Systolic blood pressure	ukb-b-20175; 4080: Output from GWAS pipeline using Phesant derived variables from UKBB (MRC-IEU)	UKBB	436,419	Hemani, 2018 [15]
Diastolic blood pressure	ukb-b-7992; 4079: Output from GWAS pipeline using Phesant derived variables from UKBB (MRC-IEU)	UKBB	436,424	Hemani, 2018 [15]
C-reactive protein level	NA	UKBB	343,524	Hemani, 2018 [15]
Liver enzyme/metabolite				
Alanine aminotransferase	ukb-d-30620_irnt	UKBB	NA	Hemani, 2018 [15]
Alkaline phosphatase	ukb-d-30610_irnt	UKBB	NA	Hemani, 2018 [15]
Aspartate aminotransferase	ukb-d-30650_irnt	UKBB	NA	Hemani, 2018 [15]
Gamma glutamyltransferase	ukb-d-30730_irnt	UKBB	NA	Hemani, 2018 [15]
Direct bilirubin	ukb-d-30660_irnt	UKBB	NA	Hemani, 2018 [15]
Total bilirubin	ukb-d-30840_irnt	UKBB	NA	Hemani, 2018 [15]
Cardiometabolic disease				
Coronary artery disease	ebi-a-GCST005195	CARDIoGRAMplusC4D and UKBB	122,773/424,528	van der Harst, 2018 [16]
Coronary artery disease	I9_IHD	FinnGen consortium	25,366/151,533	NA [21]
Heart failure	NA	HERMES (incl. UKBB)	41,734/930,298	Shah, 2020[24]
Heart failure	I9_HEARTFAIL	FinnGen consortium	9576/159,286	NA [21]

Atrial fibrillation	ebi-a-GCST006414	NA (incl. UKBB)	60,620/970,216	Nielsen, 2018 [17]
Atrial fibrillation	I9_AF	FinnGen consortium	17,325/97,214	NA [21]
Ischemic stroke	NA	MEGASTROKE	34,217/406,111	Malik, 2018 [18]
Ischemic stroke	ukb-d-I9_STR_EXH	UKBB	3314/357,880	Hemani, 2018 [15]
Ischemic stroke	I9_STR_EXH	FinnGen consortium	8046/164,286	NA [21]
Venous thromboembolism	ukb-d-I9_VTE	UKBB	4620/356,574	Hemani, 2018 [15]
Venous thromboembolism	I9_VTE	FinnGen consortium	6913/169,986	NA [21]
Type 2 diabetes	NA	DIAMANTE (incl. UKBB)	74,124/824,006	Mahajan, 2018 [19]
Type 2 diabetes	E4_DM2_STRICT (exclude type 1 diabetes)	FinnGen consortium	22,338/148,190	NA [21]
Other outcomes				
Alzheimer's disease	NA	Consortia‡ and UKBB	71,880/383,378§	Jansen, 2019 [22]
Parental lifespan	NA	LifeGene and UKBB	639,143	Timmers, 2019 [23]

CARDiOGRAMplusC4D, Coronary ARtery DIsease Genome wide Replication and Meta-analysis plus Coronary Artery Disease Genetics; DIAMANTE, DIAbetes Meta-ANalysis of Trans-Ethnic association studies; GIANT, Genetic Investigation of ANthropometric Traits; GLGC, Global Lipids Genetics Consortium; HDL, high-density lipoprotein; HERMES, Heart Failure Molecular Epidemiology for Therapeutic Targets; ICBP, International Consortium of Blood Pressure; LDL, low-density lipoprotein; MAGIC, Meta-Analyses of Glucose and Insulin-related traits Consortium; NA, not available; UK Biobank, UKBB.

*The genetic association summary statistics estimates were extracted from the MR-Base platform [15], publicly available genetic consortia data, or the last public release (R4) of the FinnGen consortium [21].

†Estimated nutrient intake based on UKBB participants' answers to the dietary questionnaire.

‡Three independent consortia, including Alzheimer's disease working group of the Psychiatric Genomics Consortium (PGC-ALZ), the International Genomics of Alzheimer's Project (IGAP), and the Alzheimer's Disease Sequencing Project (ADSP).

§The dataset consists of 24,087 clinically diagnosed late-onset Alzheimer's disease cases, paired with 55,058 controls plus the AD-by-proxy phenotype, based on individuals in the UK Biobank (UKB) for whom parental Alzheimer's disease status was available (proxy cases = 47,793; proxy controls = 328,320).
